# Towards sustainable biocontrol: inhibition of soil borne fungi by microalgae from harsh environments

**DOI:** 10.3389/fmicb.2024.1433765

**Published:** 2024-07-15

**Authors:** Dikla Eckstien, Noga Maximov, Nofet Margolis, Hagai Raanan

**Affiliations:** Department of Plant Pathology and Weed Research, Agricultural Research Organization, Institute of Plant Protection, Gilat Research Center, Rishon LeTsiyon, Israel

**Keywords:** antifungal activity, biocontrol, biological soil crusts, desmodesmus, microalgae, soilborne pathogens

## Abstract

Using microorganisms as biocontrol agents against soilborne plant pathogens is a promising alternative to chemical pesticides. However, only some biocontrol agents have proven effective under field conditions. This study explores the potential of highly resilient microalgae isolated from harsh environments, such as Biological Soil Crusts and agricultural fields in semi-arid regions, as a novel and sustainable approach to biocontrol. Fifty-nine microalgal strains, including thirteen cyanobacteria and forty-six green algae, were isolated and identified. Dual-culture plate assays and toxicity tests of microalgal growth media were conducted to evaluate the antifungal activity of the isolates against eight representative soilborne pathogens. The results showed that many microalgae strains exhibited significant inhibitory effects on the growth of specific fungal pathogens, although the activity varied among different microalgal strains and pathogen species. Some strains even promoted the growth of certain fungi. The lack of a clear pattern in the antifungal activity highlights the complexity and specificity of the interactions between microalgae and soilborne pathogens. An “Inhibition Effectiveness” metric was developed to quantify biocontrol potential based on fungal growth inhibition. The green algal genus *Desmodesmus*, particularly *Desmodesmus subspicatus* isolates, showed higher antifungal efficacy than other genera. While the inhibitory mechanisms remain unclear, the results demonstrate the promising biocontrol capabilities of microalgae from extreme environments like BSCs. Further research could unlock novel opportunities for sustainable disease management by harnessing specific microalgal strains or synergistic strain combinations targeting soilborne pathogens.

## 1 Introduction

The use of microorganisms as biocontrol agents (BCAs), along with the associated bioactive compounds they produce (collectively termed biopesticides), has been studied for many years as a safer alternative treatment against soilborne plant pathogens (Mazzola and Freilich, 2017). Despite extensive research, only a small proportion of BCAs have shown promising activity under controlled conditions, and only some ultimately reach the application stage in the field (Katan, 2017). Likely, this is a result of the intricate interplay between the biocontrol agent and the environmental biotic and abiotic stressors.

To overcome the challenges of environmental conditions in BCA development and implementation processes, researchers are constantly seeking to isolate new microorganisms from extreme environments such as desert soils and high salinity environments as potential BCAs (Nübel et al., 1997; Alsharif et al., 2020; Anwer et al., 2020).

Desert regions offer farmers many natural advantages, such as long periods of sunshine, multiple growing seasons, and fewer pests and diseases. However, environmental factors, such as soil temperatures, salinity, and water potential, frequently reach extreme levels. Furthermore, extensive use of low-quality water from recycling and saline sources for irrigation, along with high temperatures and minimal precipitation, significantly impacts soil quality (Ben-Gal et al., 2006). As over 35% of the Earth’s land mass can be classified as semi-arid, arid, or hyper-arid, it is essential to develop sustainable crop protection solutions for dryland agriculture. Moreover, almost 70% of the global land cover experienced a drying trend over the last 70 years (66% between 1948 and 2005) (Huang et al., 2016). Thus, climate change, characterized by warming, desertification, and extreme weather events, is expected to increase the search for resilient BCAs.

Biological soil crusts (BSCs) represent some of the most extreme environments on our planet, and the organisms living within them have adapted to survive in harsh environmental conditions, including drying out, high temperatures, detrimental radiation, and osmotic stress (Bettina et al., 2016). These unique biofilms cover the surfaces of approximately 35% of arid and semi-arid regions (12.2% of the global terrestrial surface) (Rodriguez-Caballero et al., 2018). The crust is formed by associating soil particles with a complex community of cyanobacteria, fungi, microalgae, lichens, mosses, and liverworts (Chamizo et al., 2013). Cyanobacteria and microalgae are known to produce an extensive array of secondary metabolites, many with antibiotic activity. Several cyanobacteria and microalgae have been identified to inhibit the activity of plant pathogenic fungi, bacteria, and nematodes (Renuka et al., 2018; Lee and Ryu, 2021). Yet very little is known about the production of secondary metabolites by BSC inhabiting microalgae. Recently, Chamizo (2018) briefly reviewed the potential of soil inoculation with cyanobacteria to improve soil health and crop productivity in drylands by enhancing soil stability, soil nutrient, and moisture status, organic matter content, microbial activities, and crop growth. Nevertheless, not much is known about the biocontrol potential of desert microalgae.

This study investigates the potential of microalgae isolated from BSCs and desert agricultural soils as a novel and sustainable approach to biocontrol. We explore the possibility that these extremophilic microalgae possess unique antifungal properties against soilborne fungi, detrimental to plant growth and agricultural productivity. The research focuses on identifying BSC microalgae strains with potent antifungal activity and evaluating their inhibitory effects on a variety of soilborne fungi. The harsh desert environment selects for microbes with robust survival strategies. These adaptations could translate to resilience and effectiveness of BSC microalgae as biocontrol agents even under stressful conditions. This study explores the potential of microalgae isolated from BSCs and agricultural soils of semi-arid regions as sustainable soilborne pathogen biocontrol agents.

## 2 Materials and methods

### 2.1 Sampling procedure

In early 2022, soil samples were collected from two distinct habitats: Biological Soil Crusts (BSC) in the NW Negev region and a cultivated potato field at the Gilat Research Center. The BSC samples were sourced from four sites: Ein Hashlosha, Ein Habsor, Sayeret Shaked Park, and the Gilat region. In the potato field, soil samples were collected from the upper layer crust and the specialized zones around the potato plants, specifically the rhizosphere and geocaulosphere (tuber zone). Approximately ten grams of soil were gathered into sterile tubes from each specialized zone of five potato plants. To collect the rhizosphere soil, we separated the soil adhering to the roots into the sterile tube. Soil attached to the tuber was considered geocaulosphere soil.

### 2.2 Sample processing and microalgae isolation

Upon collection, the soil samples were suspended in 100 mL BG11 media to support the growth and enrichment of microalgae. These suspensions were incubated at 24°C in a controlled growth room with a 12 h light/dark cycle for three days to encourage microalgal proliferation. After incubation, the samples were homogenized, and three 50 μL aliquots from each suspension were plated onto fresh BG11 agar plates. The plates were then incubated under the same conditions to allow the growth of algal colonies. Algal colonies were then isolated using the standard agar plate isolation method.

### 2.3 Microalgae identification

Axenic cultures of isolated microalgae were subcultured in fresh BG11 media to obtain biomass for further analysis. Microalgae were identified through morphological observations and molecular analysis of ribosomal RNA (rRNA) genes. Morphological characteristics were examined using light microscopy (microscope type), while molecular identification involved PCR amplification and sequencing of 16S and 18S regions of the rRNA genes to determine the taxonomic affiliation of the isolated microalgae. The primers used for the identification were SS5F- GGTGATCCTGCCAGTAGTCATATGCTTG SS3R GATCCTTCCGCAGGTTCACCTACGGAAACC for 18S (Matsumoto et al., 2010) and CYA359F—GGGGAATYTTCCGCAATGGG CYA781R—GACTACWGGGGTATCTAATCCC for cyanobacterial 16S (Nübel et al., 1997).

### 2.4 Microorganisms maintenance

Axenic cultures of isolated microalgae obtained from the soil samples were grown in BG11 medium at 25°C with a light intensity of 50 μmol photons/m^2^ for 12 h/day. Fungal soilborne pathogens, including *Verticillium dahliae, Alternaria solani, Fusarium* sp., *Rhizoctonia solani* AG3 and AG4, *Colletotrichum coccodes, Macrophomina phaseolina*, and *Sclerotium Rolfsii* from our collection at the Gilat Center for Arid and Semi-Arid Agricultural Research, Israel, were used for the experiments. The fungi were inoculated on Potato Dextrose Agar (PDA) plates and kept in a 25°C incubator for 5 days to grow before use.

### 2.5 Antifungal activity assays

To evaluate the antagonistic activity of the isolated microalgal strains against soilborne pathogens, dual-culture plate assays were conducted. Each microalgal strain was co-cultured with each of the eight representative soilborne pathogens. First, we inoculated the microalgae strain in a line in the center of a Petri dish containing PDA medium and incubate it for a week to establish. Then, we carefully inoculated a plug of each fungal pathogen onto the opposite sides of the dish, ensuring proper isolation between the two cultures. The co-culture plate assays were incubated at 25°C for another week, allowing the microalgal strains and soilborne pathogens to interact. After the incubation period, the plates were examined for any signs of antagonism, including growth inhibition or changes in colony morphology. The fungal colony area was measured to assess growth inhibition or promotion. The ratio between the co-cultured colony area and the control colony area was calculated to produce an inhibition score. Each fungus was tested in two plates, each plate with two samples.

Several microalgal strains, predominantly filamentous cyanobacteria, exhibited slow growth rates and were not conducive to the traditional dual-culture assay. Thus, we evaluated the toxicity of their growth media against the selected fungal pathogens to assess their potential antagonistic activity. Thirty-day-old axenic cultures of microalgae strains were centrifuged at 4000 rpm for 10 min to remove cell biomass. The spent media was then mixed with Potato Dextrose Broth (PDB) in a 1:1 ratio, while the control consisted of PDB and BG11 in a 1:1 ratio. Each well of a 24-well plate contained 2 mL of the spent media or control mix. Agar plugs (1 mm) covered with actively growing mycelium of the tested fungi were placed in each well, and the plate was incubated for one week at 25°C. Each fungus was tested using three replicates.

### 2.6 Inhibition effectiveness score

To quantitatively assess the biocontrol potential of various algae strains against a spectrum of fungal pathogens, we developed a novel metric termed “Inhibition Effectiveness.” This metric was derived from experimental data, including the fungal growth response ratios in the presence of different algae strains (inhibition score). The “Inhibition Effectiveness” score for each strain was calculated by aggregating the inhibitory effects across eight fungi types. The calculation method specifically focused on the inhibitory interactions, where any inhibition scores below 1 (indicating a reduction in fungal growth compared to control) contributed positively to the score. Each of these inhibition scores were summed to form a strain’s total “Inhibition Effectiveness” score. Conversely, any inhibition scores above one were assigned a score of 0, as these do not contribute to fungal inhibition. This methodological approach allows for a focused evaluation of each strain’s biocontrol efficacy, providing a clear and systematic metric for comparison.

### 2.7 Statistical analysis

The data from the antifungal screening assays were analyzed using various statistical methods. Analysis of variance (ANOVA) was employed to evaluate differences in inhibition effectiveness among microalgal genera. Pairwise comparisons using post-hoc tests (Tukey HSD Test) were conducted to identify significant differences between specific genera pairs. The inhibition effectiveness of the genus *Desmodesmus* was also compared against a combined group containing all other genera using ANOVA. Principal component analysis (PCA) was performed to visualize relationships and potential clustering patterns among the microalgal strains based on their antifungal activity profiles against the various fungal pathogens.

## 3 Results

### 3.1 Microalgae isolation

In early 2022, we gathered soil samples from Biological Soil Crust habitats in the NW Negev region and a potato field at the Gilat Research Center. The soil crusts were collected from Ein Hashlosha, Ein Habsor, Sayeret Shaked Park, and Gilat. Additionally, soil from the potato field was obtained from both the upper layer crust and the rhizosphere and geocaulosphere (tuber zone) of potato plants. Subsequently, these soil samples were incubated in BG11 media to isolate microalgae. The isolated microalgae were cultured and identified through morphology and rRNA gene analysis.

**Figure 1:**
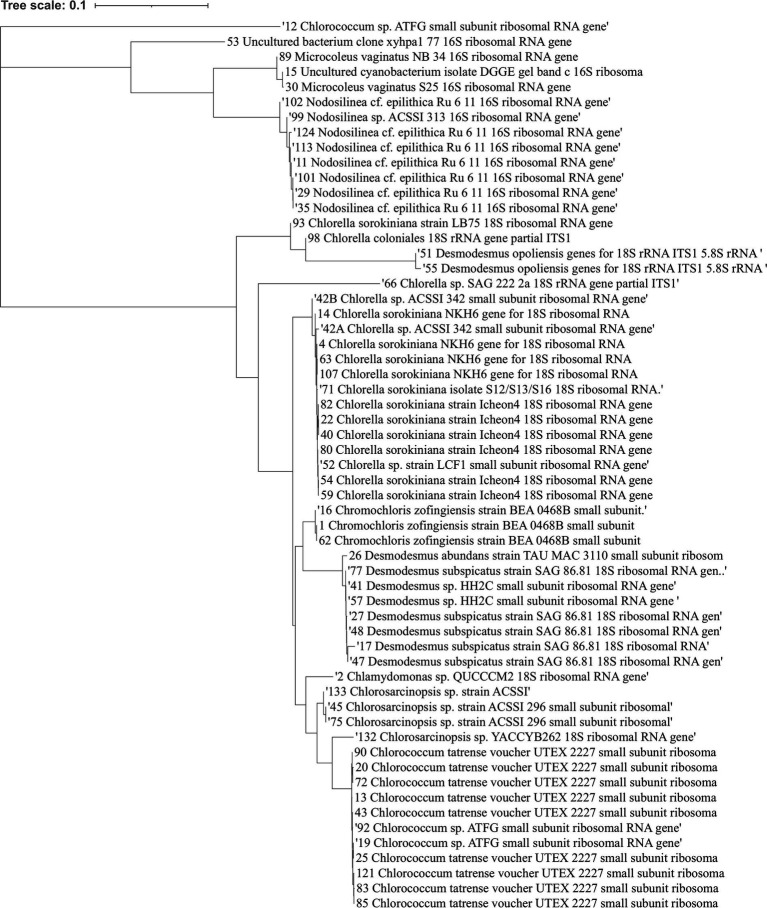
Phylogenetic tree showing the relationships between various isolated microalgal strains. The phylogenetic tree depicts the taxonomic affiliations of the 59 microalgal strains isolated in this study. The strains include 13 cyanobacteria (predominantly from the genera *Nodosilinea and Microcoleus*) and 46 green algae (from genera such as *Chlorella, Chlorococcum, Chlorosarcinopsis, Chromochloris, Desmodesmus*, and *Chlamydomonas*). The numbers alongside the strain names correspond to the strain IDs provided in Supplementary Table S1.

We successfully isolated and identified 59 microalgal strains from the collected soil samples. These included 13 cyanobacteria strains and 46 green algae strains. The cyanobacteria were predominantly from the genera *Nodosilinea* and *Microcoleus*, while the green algae were from the genera *Chlorella*, *Chlorococcum*, *Chlorosarcinopsis*, *Chromochloris*, *Desmodesmus*, and *Chlamydomonas*. A phylogenetic tree depicting these isolated microalgae can be found in Figure 1, and a complete list of the isolates is provided in Supplementary Table S1.

### 3.2 Screening for antifungal activity

**Figure 2:**
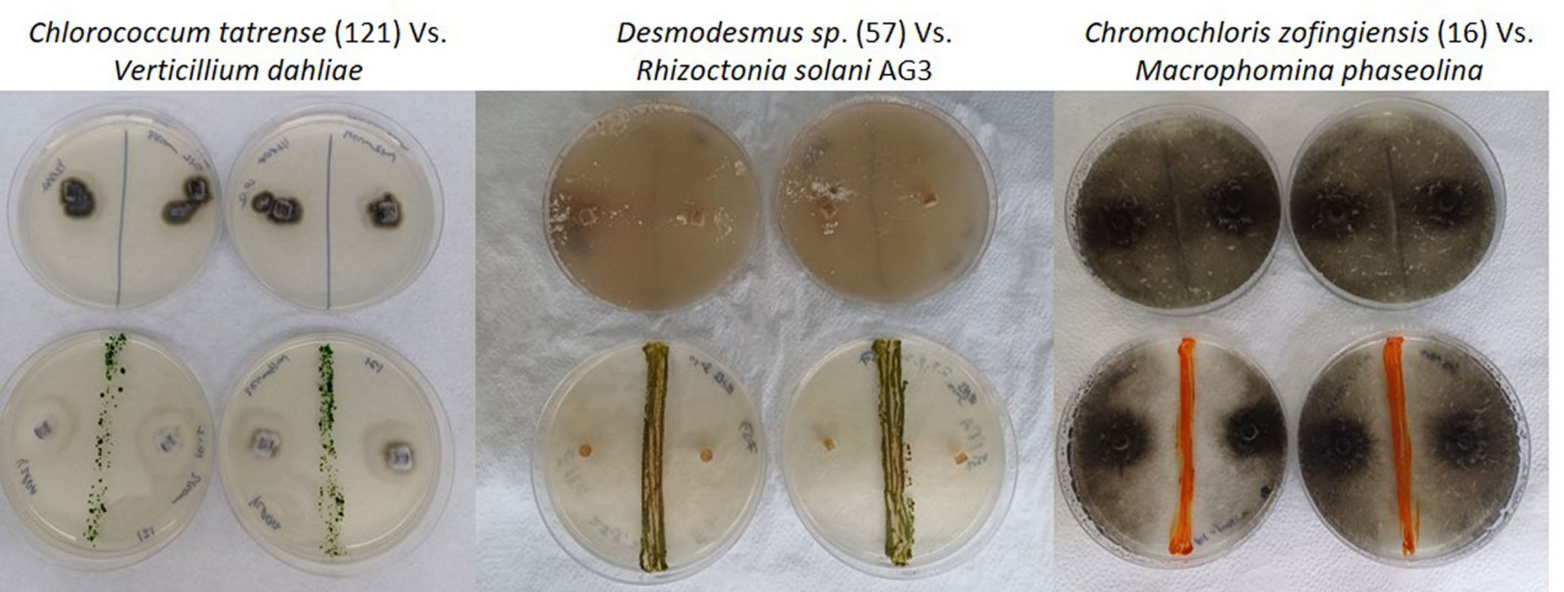
Examples of the antifungal activity exhibited by microalgae isolate strains against different soilborne fungal pathogens in dual culture plate assays. The image displays Petri dish plates from the dual culture antifungal assays, with the microalgal strains initially inoculated as a line in the center and the fungal pathogens inoculated on the opposite side. The first row displays control plates with only the fungal pathogens growing in the absence of any microalgal strains. The second row shows co-culture plates where the microalgal strains were inoculated alongside the fungal pathogens, allowing for observation of potential inhibitory effects or interactions between the two organisms. Note the loss of dark pigmentation of *Verticillium* dahlia and Rhizoctonia solani AG3 in the presence of the algae.

After isolating the microalgae strains, we conducted a screening to evaluate their antifungal activity against soilborne pathogens. We performed dual-culture plate assays, where each microalgal strain was co-cultured with eight representative soilborne pathogens, including *Verticillium dahliae, Alternaria solani, Fusarium sp., Rhizoctonia solani* AG3 and AG4, *Colletotrichum coccodes, Macrophomina phaseolina*, and *Sclerotium Rolfsii*. Figure 2 shows examples of the dual culture experiments. Some microalgae, mostly filamentous cyanobacteria, did not work well in the classical co-culture experiment due to their slow growth rate. Therefore, we tested the toxicity of their growth media on the fungi.

**Figure 3:**
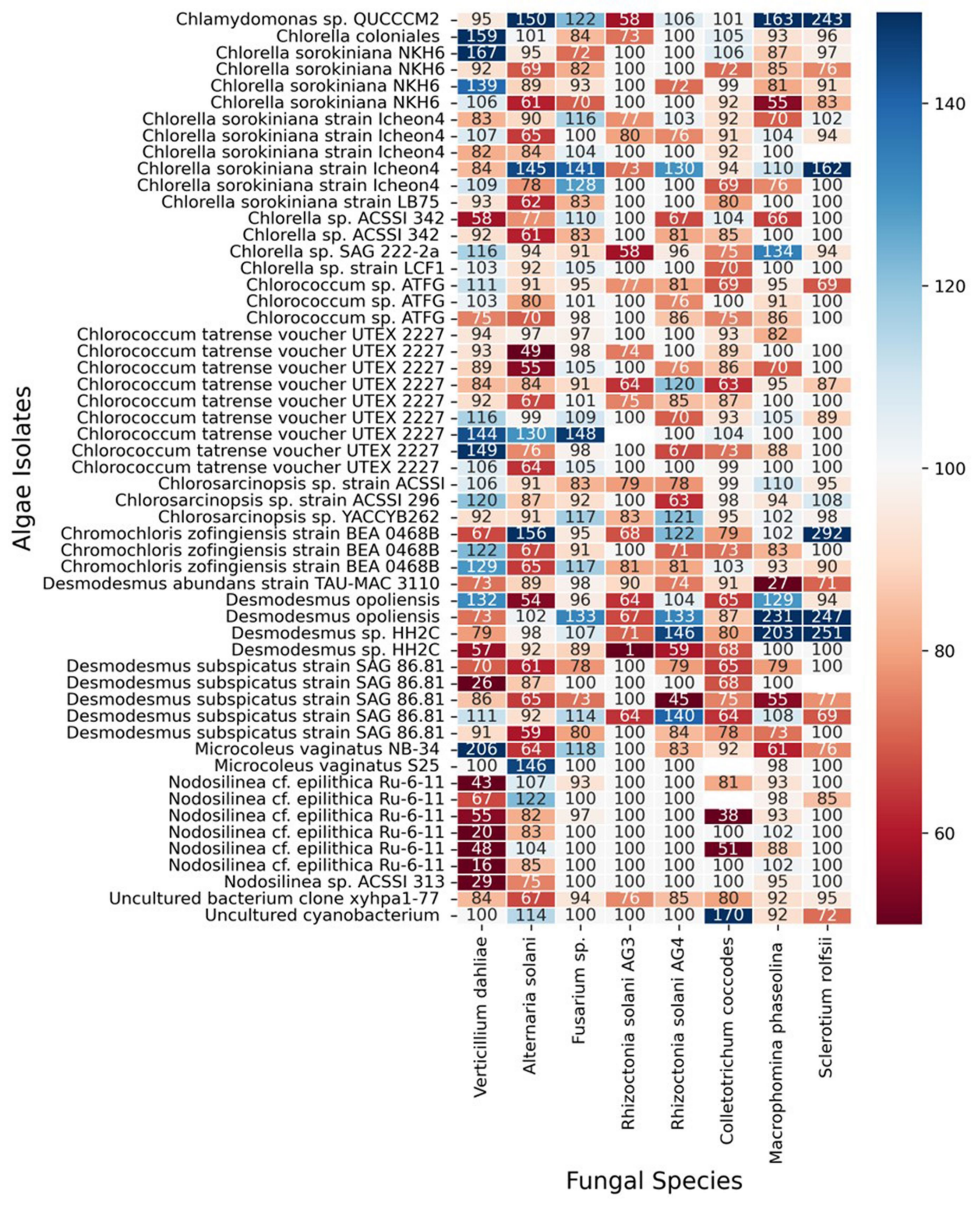
Heat map of antifungal activity of various microalgal strains against different soilborne fungal pathogens. The heat map depicts the results of the screening for antifungal activity, where each row represents a microalgal strain and each column represents a soilborne fungal pathogen tested. The numeric values in the cells indicate the percent growth inhibition or enhancement (positive or negative, respectively) of the fungal pathogens when co-cultured with the corresponding microalgal strain (inhibition score). The color scale ranges from dark red (strong inhibition) to dark blue (strong enhancement) of fungal growth.

The results of our screening showed that many microalgae strains exhibited significant antifungal activity against some of the tested soilborne pathogens. However, the antifungal activity varied among different microalgal strains and pathogen species. Moreover, some of the algal strains increased the growth and proliferation of certain fungi species. Figure 3 shows a heatmap of the effect of microalgal strains on the development of the tested fungi. Interestingly, in many cases, we observed an effect on the density and color of the fungal colony regardless of any impact on the colony size. Out of the 55 microalgae strains tested in the screening, 50 showed inhibitory effects (20% or more in growth) on at least one of the tested soilborne pathogens. At the same time, only five exhibited significant inhibition on four fungi. Notably, two isolates of *Desmodesmus subspicatus* isolated from potato plant rhizosphere inhibited six of the eight fungi. On the other hand, only 21 showed an enhancing effect (20% or more in growth) on at least one of the tested soilborne pathogens, while only three exhibited an enhancing significant impact on four fungi. Moreover, out of 556 individual microalgal-fungal interactions tested, 276 exhibited some effect on colony density or color. For example, in many cases, the fungi lost their characteristic dark pigmentation (Figure 2). This suggests a complex and dynamic interaction between microalgae and soilborne pathogens.

To quantify the biocontrol potential of the various algae strains against fungal pathogens, we developed a new metric called “Inhibition Effectiveness,” derived from experimental data on fungal growth inhibition by different algae strains. The “Inhibition Effectiveness” score for each strain was calculated by summing the inhibition scores below 1 (indicating reduced fungal growth) across eight fungi types. In contrast, scores above one were assigned 0, as they did not contribute to fungal inhibition. This metric allows for a focused evaluation of each strain’s biocontrol efficacy, providing a systematic basis for comparison.

**Figure 4:**
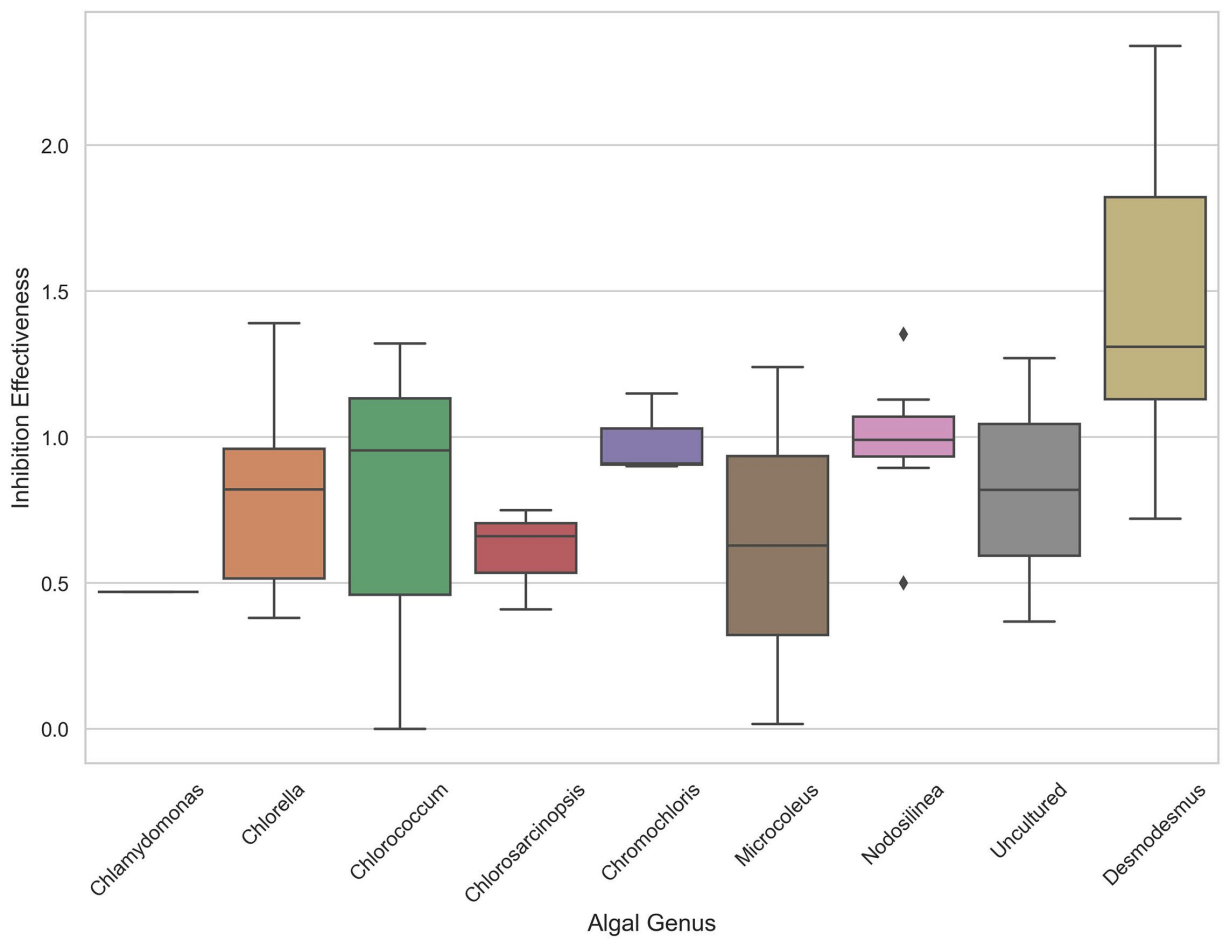
The distribution of inhibition effectiveness across various algal genera. Boxplot showing the distribution of inhibition effectiveness across various algal genera.

The analysis of inhibition effectiveness among various algal genera revealed that *Desmodesmus* stands out as a particularly effective biocontrol agent. Table 1 presents the ten most effective microalgae strains overall. Notably, the four most effective and six out of the ten most effective strains belong to the genus *Desmodesmus*. Three are the same species, *Desmodesmus subspicatus*. Statistical tests, including ANOVA and pairwise comparisons, confirmed significant differences in inhibition effectiveness between *Desmodesmus* and other genera, such as Chlorella and *Chlorococcum* (*p* < 0.05). The boxplot visualization in Figure 4 further illustrates that *Desmodesmus* shows higher median inhibition effectiveness, suggesting its robust potential in biocontrol applications. In addition to its comparison with individual algal genera, *Desmodesmus* was also evaluated against all other genera combined. The results from an ANOVA test showed a statistically significant difference (*p* < 0.01), indicating that *Desmodesmus* is more effective than all other tested algal genera. These findings highlight *Desmodesmus* as a promising candidate for further research and application in managing fungal pathogens, potentially leading to more sustainable agricultural practices.

**Table 1:** The ten most antifungal effective isolated microalgae strains.

Isolate number	Name	Inhibition effectiveness
57	*Desmodesmus* sp. HH2C	2.34
47	*Desmodesmus subspicatus* strain SAG 86.81	2.24
26	*Desmodesmus abundans* strain TAU-MAC 3110	1.87
17	*Desmodesmus subspicatus* strain SAG 86.81	1.68
14	*Chlorella sorokiniana* NKH6	1.39
101	*Nodosilinea cf. epilithica* Ru-6-11	1.35
77	*Desmodesmus subspicatus* strain SAG 86.81	1.35
42A	*Chlorella* sp. ACSSI 342	1.32
83	*Chlorococcum tatrense* voucher UTEX 2227	1.32
51	*Desmodesmus opoliensis*	1.27

**Figure 5:**
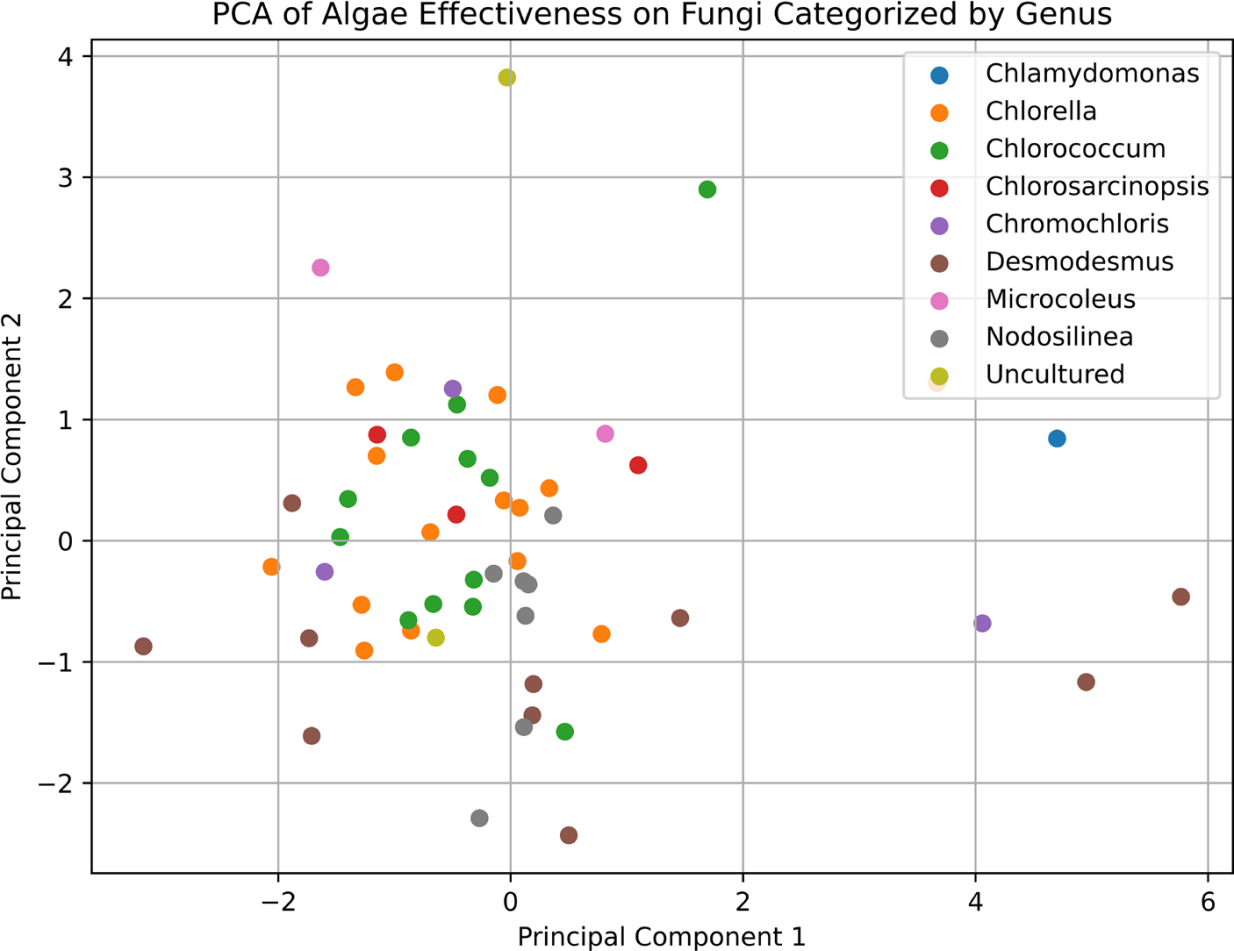
Principal component analysis (PCA) plot of the relationships between microalgal strains based on their antifungal activity against various soilborne pathogens. The PCA plot shows the distribution of the microalgal strains, which are color-coded by their taxonomic affiliations. The proximity of the points on the plot indicates the similarity in the antifungal activity profiles of the corresponding microalgal strains. The lack of clear clustering patterns suggests the highly specific and complex nature of the interactions between the microalgae and the soilborne fungal pathogens, as mentioned in the discussion section of the paper.

However, it is important to note that even the *Desmodesmus* isolates show a broader range of effectiveness between different isolates. Despite the high percentage of microalgae showing some inhibitory effect, we could not detect any pattern or commonality among the microalgal strains that exhibited antifungal activity and the inhibited soilborne pathogens, as shown in the PCA analysis presented in Figure 5. This observation highlights the high specificity of the inhibition process and the complexity of interactions between microalgae and soilborne pathogens. It also suggests the potential for using specific microalgae strains as biocontrol agents against soilborne pathogens. For example, all the *Desmodesmus* isolates had only a mild effect on *Colletotrichum coccodes*, while the best strains against it included *Nodosilinea cf. epilithica* and *Chlorococcum tatrense*. Supplementary Table S2 presents the three strains with the highest inhibition score against each pathogenic fungus.

## 4 Discussion

The results of our study highlight the potential of microalgae from Biological Soil Crusts and potato fields as sustainable soilborne pathogen biocontrol agents. The isolation and identification of 59 microalgal strains, including 13 cyanobacteria strains and 46 green algae strains, demonstrate the diverse microalgal community inhabiting these habitats. This diversity presents an exciting opportunity to develop novel biocontrol strategies in agriculture.

A key finding of this research is the significant variation in antifungal activity among different microalgal strains and species against various soilborne pathogens. This strain-level specificity highlights the complexity of microalgae-pathogen interactions and the need for a targeted approach to biocontrol strategies. Rather than relying on broad-spectrum agents, tailored combinations of specific microalgal strains could be developed to target particular pathogen species or communities more effectively. Such targeted biocontrol approaches could enhance efficacy while minimizing potential off-target effects on non-pathogenic microorganisms.

Among the microalgal genera evaluated, *Desmodesmus* emerged as a particularly promising candidate for biocontrol applications, with several strains exhibiting robust antifungal activity across multiple pathogen species. Notably, *Desmodesmus subspicatus* demonstrated broad-spectrum inhibition against six out of the eight tested fungal pathogens. *Desmodesmus* is a genus of green microalgae with a high degree of phenotypic plasticity, enabling it to thrive in various environments and withstand fluctuations (Lin et al., 2020). Recently, *Desmodesmus abundance* has been suggested as a biofertilizer for common bean fields (Lopes et al., 2023). To our knowledge, work has yet to be done on the potential of the genus *Desmodesmus* as a biocontrol agent or its antibiotic and antifungal capabilities. Most of the *Desmodesmus* strains in this work are isolated from the rhizosphere of potato plants in the Gilat research center; however, the strain with the highest inhibition effectiveness is *Desmodesmus* sp. HH2C (Number 57) was isolated from the natural crust in Sayert Shaked. This finding positions *Desmodesmus* as a promising candidate for potential applications in fungal disease management strategies. However, it is crucial to recognize that substantial variability exists among different isolates, even within this genus, necessitating careful strain selection for optimal biocontrol outcomes.

While the antifungal activity of various microalgal strains is evident from this study, the underlying mechanisms behind this biocontrol activity remain unclear. The highly specific nature of the observed microalgae-fungus interactions implies that a comprehensive understanding of these responses’ underlying mechanisms is essential for harnessing their full potential. Future research is necessary to understand the antifungal activity of each strain better and identify the bioactive compounds or metabolites responsible for the antifungal effects. Additionally, exploring potential synergistic interactions between multiple microalgal strains could unlock novel avenues for enhancing biocontrol efficacy.

Translating the promising laboratory findings into practical field applications of microalgal biocontrol agents presents several challenges and considerations. Despite thorough investigation, only a limited number of the potential BCAs successfully transition from controlled laboratory conditions to successful field applications. This is probably attributed to the intricate interplay between the biocontrol agent and its biotic and abiotic environment. Numerous publications have shown the impact of environmental conditions on the inhibitory activity of BCAs. For example, pH, moisture, temperature, and light affect the antifungal activity of *Trichoderma atroviride* on *Rhizoctonia solani* (Daryaei et al., 2016). Moreover, optimal conditions for maximal growth of BCA do not necessarily produce the best and most fit microorganism. Here, we demonstrated the potential of *Desmodesmus* strains and other microalgae isolated from harsh environments, such as the biological soil crusts, to inhibit the growth of a range of soilborne pathogens. Thus, it is likely that these microalgae isolates will perform better under environmental stress. However, the production of antifungal compounds was examined only under controlled laboratory conditions. The production of secondary metabolites by microalgae is tightly connected to environmental signals (De Morais et al., 2015) Thus, they are produced at a low abundance or not at all expressed under different environmental conditions (Rutledge and Challis, 2015; Margolis et al., 2023). Further research is needed to evaluate the antifungal activity of these strains in realistic environmental conditions. This will help optimize application conditions and increase the chances of success (Margolis et al., 2023).

## 5 Conclusion

The study successfully isolated and identified 59 soil microalgal strains from BSCs and potato fields that exhibited antifungal activity against major soilborne fungal pathogens in screening assays. The wide variation in antifungal activity among different microalgal strains and pathogen species underscores the specificity and complexity of their interactions. Of the microalgal genera evaluated, *Desmodesmus*, particularly the *D. subspicatus* isolates, emerged as the most promising biocontrol candidate based on the “Inhibition Effectiveness” metric quantifying fungal growth inhibition. This positions *Desmodesmus* as a strong focus for further research into harnessing microalgae as biocontrol agents.

While the study provides valuable insights, further investigation is needed into the mechanisms underlying the antifungal activity, the specific bioactive compounds involved, and evaluating efficacy under field conditions. Optimizing application conditions and exploring synergistic strain combinations could enhance biocontrol effectiveness for real-world applications in agriculture.

## Data availability statement

The datasets presented in this study can be found in online repositories. The names of the repository/repositories and accession number(s) can be found in the article/Supplementary material.

## Author contributions

DE: Investigation, Writing – review & editing. NgM: Investigation, Writing – review & editing. NfM: Investigation, Writing – review & editing. HR: Conceptualization, Formal analysis, Funding acquisition, Methodology, Supervision, Writing – original draft, Writing – review & editing.
